# A web-based psycho-educational intervention (Fex-Can) targeting sexual dysfunction and fertility-related distress in young adults with cancer: study protocol of a randomized controlled trial

**DOI:** 10.1186/s12885-019-5518-3

**Published:** 2019-04-11

**Authors:** C. Lampic, L. Ljungman, C. Micaux Obol, L. E. Eriksson, L. Wettergren

**Affiliations:** 1 Department of Women’s and Children’s Health, Karolinska Institutet, Solna, Sweden; 2 Department of Learning, Informatics, Management and Ethics, Karolinska Institutet, SE-171 77 Stockholm, Sweden; 3 School of Health Sciences, City, University of London, London, EC1V 0HB UK; 4 Department of Infectious Diseases, Karolinska University Hospital, SE-141 86 Huddinge, Sweden

## Abstract

**Background:**

This study protocol describes the clinical trial of the Fex-Can intervention, a web-based self-help program targeting sexual dysfunction and fertility-related distress. The psycho-educational intervention has been developed in collaboration with young patients with cancer and shown to be feasible. The primary objective is to determine whether the Fex-Can intervention, provided in addition to standard care, is superior to standard care in terms of reduction of sexual dysfunction and fertility-related distress directly after end of the 12-week program. The trial also aims to determine whether the intervention has an effect on the secondary outcomes including health-related quality of life, anxiety, depression, body image, fertility knowledge, and self-efficacy related to sexuality and fertility.

**Methods:**

The trial has an randomized clinical trial (RCT) design with two parallel arms. The active groups receive either the version of the Fex-Can intervention targeting sexual problems or the version targeting fertility-related distress. Control groups receive standard care. Primary outcomes will be sexual function assessed with the Patient-Reported Outcomes Measurement Information System® Sexual Function and Satisfaction measure version 2.0 (SexFS) and fertility-related distress assessed with the Reproductive Concerns After Cancer scale (RCAC). The effect of the intervention will be evaluated directly after end of the program. Primary and secondary outcomes will also be assessed at the short- (12 weeks after end of program) and long-term (20 and 44 months after end of program) follow-up. At least 64 completers will be needed in each arm (total *n* = 256) to achieve adequate statistical power in the analyses. In order to increase the understanding of how the intervention brings about a possible change, semi-structured interviews will additionally be conducted with a purposeful sample shortly after completion of the intervention.

**Discussion:**

If the Fex-Can intervention proves to be efficacious the necessary steps will be taken to implement it in routine care for young adults diagnosed with cancer. Healthcare could thereby be provided with an easily accessible, cost-effective intervention to offer to young adults suffering from fertility-related distress or sexual problems.

**Trial registration:**

ISRCTN36621459. Registered 25 January 2016.

## Background

### Background and rationale

Sexual dysfunction and fertility-related distress among young adults are common in the aftermath of cancer. Previous research has shown that about 50% in this population report sexual dysfunction one year after the cancer diagnosis [1]. Reduced sexual desire, dyspareunia, vaginal dryness and low satisfaction with sex life are commonly reported by women [2, 3], whereas erectile dysfunction [4], orgasmic difficulties [5] and reduced sexual interest [6] are reported by men. Several of the most common cancer treatments (radiation therapy, chemotherapy, endocrine treatment) may cause these problems directly or indirectly via physiological, psychological, and interpersonal factors [7]. These treatments may also cause temporary or permanent infertility or subfertility [8]. A majority of young women with cancer report fertility-related distress at clinical levels, which also is related to reduced quality of life and to long-term depressive symptoms [9]. Men’s experiences of fertility-related distress after cancer have been studied to a lesser extent and more research is needed to establish the prevalence of clinical levels of distress by cancer type. Importantly, young people with cancer have themselves ranked both sexual problems and fertility concerns as among their core needs that are unmet by the healthcare today [10–12].

e-Health interventions have the potential to improve quality of life and other behavioral outcomes by supporting participants’ autonomy, competence, and relatedness [13]. While psychosocial interventions are increasingly being used in young adults with cancer [14], web-based interventions to overcome sexual and reproductive problems are mostly lacking. One program providing information about reproductive health and fertility following cancer has shown positive effects in terms of social and physical functioning, and improved fertility knowledge [15]. Also, a web-based intervention to alleviate sexual problems among women with cancer showed positive results over time, but the participants were all 35 or older [16]. These results indicate that e-health interventions have the potential to contribute to improved sexual and reproductive health in cancer populations, but that specific attention should be given to the preferences and needs of young people with cancer.

In order to address the problems mentioned above, we initiated the project Fertility and Sexuality following Cancer (Fex-Can). Within this project a web-based intervention has been developed to fill the gap of evidence-based psychosocial interventions to cope with sexual dysfunction and fertility-related distress in young people diagnosed with cancer. The Fex-Can intervention has been developed in close collaboration with young patients with cancer and significant others [17]. If the intervention proves to be efficacious, it can contribute to improved sexual and reproductive health in young adults with cancer.

The Fex-Can project, besides the development and evaluation of the Fex-Can intervention, includes a population-based cohort study (Fex-Can Cohort) monitoring sexual dysfunction and fertility-related distress in young adults with cancer over 5 years following diagnosis. The Fex-Can intervention is evaluated in a randomized controlled trial (RCT) embedded in the Fex-Can Cohort. The current protocol describes the RCT of the Fex-Can intervention.

### Objectives

The primary objective of the current trial is to determine whether the Fex-Can intervention, provided in addition to standard care, is superior to standard care in terms of reduction of sexual dysfunction and fertility-related distress directly after end of the program. The trial also aims to determine whether the intervention has an effect on the secondary outcomes including health-related quality of life, anxiety, depression, body image, fertility knowledge, and self-efficacy related to sexuality and fertility.

### Trial design

**Fig. 1:**
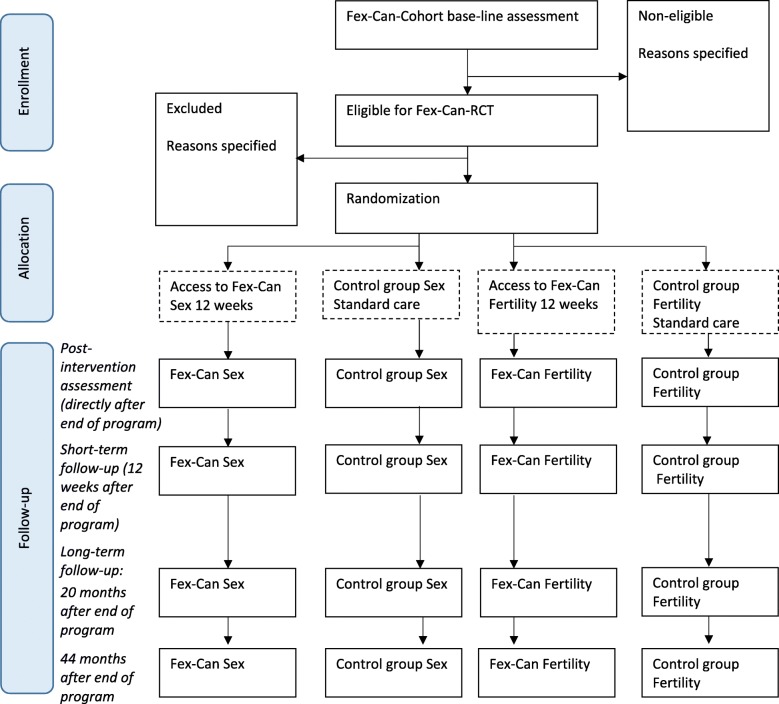
CONSORT standard RCT flow diagram (numbers, where they appear, are estimates at this stage)

The trial will have a two parallel-group pre-post and follow-up (short- and long-term) superiority randomized controlled design with a 1:1 allocation ratio. The active groups will receive the Fex-Can Sexuality program (Fex-Can Sex) or the Fex-Can Fertility program (Fex-Can Fertility) according to level of self-reported problems. The intervention, Fex-Can Sex or Fex-Can Fertility, will be compared with active control groups who receive standard care, as shown in the Consolidated Standards of Reporting Trials (CONSORT) flow diagram (Fig. 1). This study protocol adheres to the SPIRIT statement for clinical trial protocols [18, 19] and the SPIRIT-PRO Extension [20].

## Methods

### Study setting

The sample for the Fex-Can RCT will be drawn from participants at the baseline assessment of the Fex-Can Cohort, which is described in detail in a separate protocol. The Fex-Can Cohort investigates sexual dysfunction and fertility-related distress in persons diagnosed with selected cancers at the age of 18–39 over a period of 5 years following diagnosis. The diagnoses included in the Fex-Can Cohort are selected on the basis that the diseases and/or their treatments may have negative consequences for fertile ability and sexual life. Participants in the Fex-Can Cohort are identified via the Swedish National Quality Registries for Brain Tumors, Breast Cancer, Gynecological Oncology, Lymphoma, and Testicular Cancer. Approximately 1500 individuals will be approached for the Fex-Can Cohort and we estimate that about 1050 (70%) individuals will participate in the baseline assessment by completing a survey (web or paper) approximately 1.5 year after diagnosis.

### Eligibility criteria

The following inclusion criteria will be used for the Fex-Can RCT:

1. Diagnosed with malignant brain tumor, breast cancer, cervical cancer, lymphoma, ovarian cancer, or testicular cancer when aged 18–39 years approximately 1.5 years previously and subsequently included in the Fex-Can Cohort.
2. Reporting sexual dysfunction or a high level of fertility-related distress at the baseline assessment of the Fex-Can Cohort. To be eligible for the Fex-Can Sex program, individuals should report sexual dysfunction (defined as 0.5 SD from the population mean) in at least one of the selected domains of the Patient-Reported Outcomes Measurement Information System® Sexual Function and Satisfaction measure version 2.0 (SexFS version 2) [21]. To be eligible for the Fex-Can Fertility program, individuals should report a high level of fertility distress (defined as a mean score > 3) in at least one dimension of the RCAC (for further specification of assessments, se primary outcomes section below) [22]. In the event that individuals meet the eligibility criteria for both programs, their individual scores will be evaluated with regard to severity of reported problems by a registered psychologist and a registered nurse specialized in psychosocial oncology. Based on a joint decision these individuals will be allocated to one of the programs accordingly.

The following exclusion criteria will be used for the Fex-Can RCT:

1. Non-ability to read and/or understand Swedish language.
2. Reporting poor health and/or significant cognitive impairment that hinder participation in the intervention.

### Intervention

The Fex-Can intervention consists of two programs, The Fex-Can Sex and the Fex-Can Fertility, targeting sexual dysfunction and fertility-related distress respectively. The web-based self-help program has been developed in accordance with theory [23] and through a co-creative long-term collaborative process with patient research partners described in a previous publication [17]. The feasibility of the intervention has been assessed with satisfying results indicating it to be suitable for young persons with cancer regarding demand, acceptability, preliminary efficacy, and functionality [24].

Both programs, the Fex-Can Sex and the Fex-Can Fertility, are organized in six consecutive modules which will be delivered over a period of 12 weeks. The content of the modules has been described in more detail in the previous publication by Wiklander et al., [24]. The modules include information with educational and behavior change content, exercises, illustrations, quizzes, and video vignettes with young adults with cancer describing their experiences regarding the topics addressed. The exercises aim at increasing sexual pleasure and functioning (Fex-Can Sex) and handling threatened or lost fertility (Fex-Can Fertility). The intervention also includes an online-moderated joint discussion forum. In addition, the sexuality program offers voluntary telephone consultations at the beginning and end of the program aiming to orientate the participant in terms of how the program can be used and tailored to their specific needs. The intervention is delivered via a platform created with a responsive design which can be used on computers, tablets, and smartphones.

### Adherence

To enhance participants’ adherence to the intervention notifications by text message and/or e-mail will be sent at the opening of each of the six modules.

Adherence will mainly be evaluated by the log function in the web platform, recording all information about participants’ log-ins and use of different program functions. Furthermore, the post-intervention assessment (directly after end of program) will include questions about the participants’ use of different program features in terms of frequency and intensity.

### Concomitant care

Concomitant care and treatments for sexual dysfunction and/or fertility-related distress will not be prohibited by study participation.

### Measurements

The standardized measures included in the study will be used and analyzed in accordance with their respective manual. The package of questionnaires has been tested in two groups of young women and men with cancer and showed to be well accepted [3]. See Table 1 for administration of instruments.

**Table 1:** Timeline for Fex-Can RCT

	Baseline (1.5 years after diagnosis)	Post-intervention assessment (directly after end of program)	Short-term follow-up (12 weeks after end of program)	Long-term follow-up (20 months after end of program)	Long-term follow-up (44 months after end of program)
Survey data					
SexFS	X	X^a^	X^a^	X	X
RCAC	X	X^b^	X^b^	X	X
HADS	X	X	X	X	X
EORTC QLQ-30	X	X	X	X	X
BIS	X	X	X	X	X
Self-efficacy Sex	X	X^a^	X^a^	X	x
Self-efficacy Fert	X	X^b^	X^b^	X	X
Fertility related knowledge	X	X^b^	X^b^	X	X
Clinical data					
Diagnosis, type and stage	X				
Cancer treatment	X				
Semi-structured interview		X			

^a^PROMIS SexFS and Self-efficacy Sex is completed by participants in the Fex-Can Sexuality program; ^b^The RCAC, Self-efficacy Fert and Fertility related knowledge is completed by participants in the Fex-Can Fertility program. Base-line assessment is collected at approximately one year after diagnosis and follow-up assessment is based on time after end of program

### Primary outcomes

#### Sexual function

The SexFS (version 2) is a set of measures developed to assess sexual function and satisfaction in both men and women, regardless of sexual orientation and if currently being sexually active with partner/s or not. The primary outcome for the Fex-Can Sex program is the score (continuous measure) in the domain ‘Satisfaction with sex life’. This domain assesses how satisfying and pleasurable current sexual activities are perceived; two selected items will be used that are scored on a five-point scale (ranging from 1 = None/Not at all to 5 = Very/A lot). Item response theory is used to calculate the domain score which is transformed to a T-score metric where 50 represents the mean for the American general population (standard deviation = 10) [21]. Sexual dysfunction is defined as 0.5 SD (5 points on the T-scale) from the population mean of 50 in the respective domain. The SexFS has shown adequate content, construct and known-groups validity as well as test-retest reliability [21, 25]. The selected items and domains of the SexFS have been translated into Swedish and linguistically validated in accordance with the procedure developed by FACITrans and PROMIS [26].

#### Fertility-related distress

The primary outcome of the Fex-Can Fertility will be the summary score of the Reproductive Concerns After Cancer (RCAC). The RCAC is a multidimensional scale, assessing a range of fertility and parenthood concerns, developed and evaluated for young adult female cancer survivors (age 18–35 years) [22]. The scale encompasses 18 items scored on a five-point scale (ranging from 1 = Strongly disagree to 5 = Strongly agree) and includes six dimensions. The dimensions (consisting of three items each) of the RCAC are: Fertility potential, Partner disclosure, Child’s health, Personal health, Acceptance and Becoming pregnant. In each dimension, high level of reproductive concerns is defined as a mean score > 3. The RCAC has demonstrated satisfactory internal consistency and construct validity [9]. The scale has independently been translated into Swedish by two bilingual researchers, and has been evaluated by one bilingual panel (*n* = 4), one lay panel (*n* = 7) and one patient panel (*n* = 8), as well as by cognitive interviews with 3 young patients with cancer before the launch of the Fex-Can project.

### Secondary outcomes

The secondary outcomes include additional domains of sexual function (for participants in the Fex-Can Sex) and fertility-related knowledge (for participants in the Fex-Can Fertility). In addition, body image, health-related quality of life, anxiety and depression, self-efficacy (related to sexuality or fertility) measures will be used as secondary outcomes in both arms.

#### Sexual function

The following domains in the SexFS version 2 will be used as secondary outcomes: Interest in sexual activity (2 items), Bother regarding sexual function (4 items), Orgasm-ability (1 item), Orgasm-pleasure (1 item), Vaginal lubrication (women, 2 items), Vaginal discomfort (women, 4 items), Vulvar discomfort – Labia (women, 1 item), Vulvar discomfort – Clitoral (women, 1 item), and Erectile Function (men, 3 items). Domain scores are transformed to a T-score metric where 50 represents the mean for the American general population (standard deviation = 10) [21]. Additionally, sexual activity screeners from the SexFS will be used.

#### Body image

Body image will be assessed using the Body Image Scale (BIS) that measures body image discomfort associated with cancer and cancer treatment [27]. The BIS encompasses 10 items and has shown high test-retest reliability and good internal consistency in cancer patients [27].

#### Health-related quality of life

Health-related quality of life will be measured using the EORTC QLQ-C30 (version 3.0), which is a 30-item questionnaire developed to assess the quality of life of cancer patients [28, 29]. The EORTC QLQ-30 (v 3.0) includes five functional scales, three symptom scales, a global health status scale, and six single items and has demonstrated good psychometric properties in cancer populations [28, 30]. The summary score for the QLQ-30 will be used according to the EORTC QLQ-C30 Scoring Manual (3rd Edition) (2001) and Geisinger et al. [29].

#### Anxiety and depression

Anxiety and depression will be assessed using the Hospital Anxiety and Depression scale (HADS) [31]. The HADS consists of two 7-item subscales, one measuring symptoms of anxiety and the other symptoms of depression. The internal consistency of the subscales has been reported to be satisfactory and the concurrent validity has been reported to be good to very good [32].

#### Self-efficacy

Self-efficacy will be assessed by a study-specific questionnaire measuring confidence in one’s own ability to handle situations, thoughts and emotions related to the threat of infertility (6 items) and sexuality (6 items). Examples of questions assessing self-efficacy are “I feel confident that I can handle negative thoughts and emotions in relation to my sex life” (Fex-Can Sex) and “I feel confident that I can handle negative thoughts and emotions related to my reproductive ability” (Fex-Can Fertility). Mean sum scores range from 6 to 24, with higher scores indicating higher levels of self-efficacy related to sexuality and fertility, respectively.

#### Fertility-related knowledge

Fertility-related knowledge will be examined by a study-specific questionnaire with 10 items measuring perceived level of knowledge about general and cancer-related fertility issues. Responses are given on a four-point scale ranging from 1 = Disagree completely to 4 = Agree completely. Examples of items are: “I have good knowledge regarding the menstrual cycle and when a pregnancy can occur” and “I have good knowledge regarding the effect of cancer and cancer treatments on reproductive ability”. The ratings on the 10 items are summated giving sum scores with a possible range from 10 to 40, with higher scores indicating higher levels of perceived fertility-related knowledge.

### Administration of instruments

The instruments will be administered in the following order at all assessments: BIS; RCAC; Self-efficacy Fertility; Fertility-related knowledge; Self-efficacy Sexuality; SexFS; HADS; and EORTC QLQ-30. See Table 1 for outline of the study timeline.

### Clinical data

Following formal consent from each registry, clinical data will be collected from the National Quality Registries for Brain Tumors, Breast Cancer, Gynecological Oncology, Lymphoma, and Testicular Cancer and include cancer type and clinical stage, date of diagnosis, and type of treatment. Clinical variables are selected in close collaboration with representatives from each of the respective National Quality Registry, these are the same registers that also were used for identification of potential participants for the Fex-Can Cohort.

### Process evaluation

#### Post-intervention survey

As part of the post-intervention assessment, participants in the intervention groups (Fex-Can Sex and Fex-Can Fertility) will be requested to respond to 13 study-specific items concerning their adherence, i.e. use of the different features of the intervention (e.g. texts, exercises and videos). They will also be asked to state their opinions about the content and features of the intervention. Responses are given on a four-point scale ranging from “Disagree completely” to “Agree completely”. In addition, participants will be requested to declare their current level of problems (regarding sex-life or fertility-related distress) in comparison with their level of problems before they entered the Fex-Can intervention. Responses are given on a 7-point scale, ranging from “Much improved” to “Much worsened” with the midpoint “No change”.

#### Semi-structured interview

In order to increase the understanding of how the intervention is used by participants and how it brings about a possible change in the outcomes, semi-structured interviews will be conducted with a purposive sample of approximately 30 participants shortly after completion of the intervention. During these interviews, which will be conducted via telephone and transcribed verbatim, participants’ views of their sexual dysfunction or fertility-related distress and if/how these problems have changed during the course of the intervention will be explored. Participants will also be asked to describe their experiences of participating in the intervention and their use of the different program functions. Transcripts of the interviews will be analyzed using [qualitative] content analysis [33].

### Participant timeline

The Fex-Can project includes a large population-based cohort study with an embedded RCT. This means that participants in the Fex-Can RCT will be identified among participants of the Fex-Can Cohort, based on their self-report of sexual function and fertility distress at the base-line assessment. Following randomization of those who accept participation in the RCT, the intervention groups (IG) receive access to the web-based intervention delivered over 12 weeks and the control groups (CG) receive standard care. All participants (IG and CG) will be requested to complete two assessments (on paper); directly after and 12 weeks after end of program. The long-term follow-up assessments (20 and 44 months after end of program) will be conducted as part of the data collection for the Fex-Can Cohort, and will be completed on paper or via the web. Primary and secondary outcomes of the Fex-Can RCT are measured pre-treatment (baseline assessment of the Fex-Can Cohort), at post-intervention assessment, and at the short- and long-term follow-up assessments. See Table 1 for study timeline.

### Sample size

To detect a statistically significant difference with a power of 80%, estimating medium effect size (0.5) and α = 0.05, a total of 128 completers (post-intervention assessment) will be needed in the Fert-Can Sex and Fex-Can Fertility, respectively. We expect approximately 80% (*n* = 840) of the estimated 1050 participants in the Fex-Can Cohort to rate sexual dysfunction and/or a high level of fertility distress. Of these we expect approximately 50% (*n* = 420) to agree to participation in the RCT, i.e. 210 participants in the Fex-Can Sex and the Fex-Can Fertility, respectively. We estimate an attrition of 15% at the post-intervention assessment (directly after end of program) and another 15% at the short-term follow-up (12 weeks after end of program), leaving 151 participants in the Fex-Can Sex and Fex-Can Fertility, respectively. We therefore estimate that the planned number of approached individuals will be sufficient to achieve adequate statistical power.

### Recruitment

Individuals matching the inclusion criteria for the Fex-Can RCT will be sent an information letter outlining the study procedures and the voluntary nature of participation. Based on the self-reported problems at the baseline assessment of the Fex-Can Cohort, potential participants will be invited to participate in the RCT, for either the Fex-Can Sex or the Fex-Can Fertility. Acceptance of participation in the RCT is provided by return of a written informed consent form. Two reminders will be sent to non-responders. Based on some difficulties recruiting participants to the feasibility study of the Fex-Can [24] the information letter states that participants will receive two cinema tickets (total value of approximately 20 Euro) as incentives for completion of each assessment. No incentive will be offered for participation in the post-intervention semi-structured interview.

### Randomization

Participants will be randomly assigned to either intervention or control group with an allocation ratio of 1:1, allocating participants in blocks stratified by sex and diagnosis. This process will be performed separately for the arms of the RCT (Fex-Can Sex and Fex-Can Fertility). A computer-generated randomization sequence will be created by a statistician with no clinical involvement in the trial. The details of the series of random numbers is unknown to the investigators. Following randomization, participants in the intervention groups will receive a text message and/or e-mail with log-in details to the program; individuals in the control groups will be informed about their group allocation via mail or e-mail.

### Blinding (masking)

Due to the nature of the intervention, blinding of participants to researchers involved in providing and monitoring the intervention and data collection is not possible. However, participants’ allocation to intervention or control groups will be masked in the data set available for researchers during data analysis.

### Statistical methods

Besides descriptive statistics and visualizations, the main class of statistical method used will be linear mixed models for longitudinal data. The statistical analyses will primarily focus on comparing the Fex-Can intervention with standard care between baseline and directly after end of program. Missing data will be analyzed using descriptive statistics and significance testing. Intention-to-treat will be applied for analyses of the primary outcomes. Statistical analyses will be performed in collaboration with external statisticians, who are not informed about the study participants’ group allocation. SPSS Statistics version 25 (IBM Corp., Armonk, N.Y., USA) will be the primary tool for data management and statistical analyses.

### Adverse effects

Adverse effects will be evaluated by number of participants reporting possible worsening of symptoms in the primary or secondary outcomes*.* Participants furthermore will indicate in the post-intervention survey if they have experienced worsening of symptoms of any kind. Additionally, the semi-structured post-intervention interview will include a question regarding possible adverse effects experienced by the participants.

### Ethics and dissemination

#### Research ethics approval

Ethical approval has been obtained for the study procedures by the Regional Ethical Review Board in Stockholm, Sweden (Dnr: 2013/1746–31/4; 2014/2244–32; 2017/916–32; 2017/1416–32).

#### Confidentiality

All participants will receive a unique code number indicated on the survey. The code key will be stored separate from the research data and will only be accessible by members of the research team. Participants will log in to the web portal by using an alias of their own choice. The researchers will be able to connect participants’ alias to the code number at the stage of analyzing data. All data will be handled and stored according to the EU General Data Protection Regulation (GDPR).

#### Dissemination policy

The results from the trial will be communicated to the scientific, clinical and patient communities through publications in scientific peer-reviewed open-access journals and presentations at international clinical and scientific conferences and in other contexts.

## Discussion

Numerous studies have reported sexual problems and fertility-related distress to be common among young adults following a cancer diagnosis [1, 9]. Young adults with cancer have furthermore themselves ranked both sexual problems and fertility concerns as among their core problems in the aftermath of cancer [10–12]. Still, evidence-based interventions treating these problems are lacking. The Fex-Can intervention is a web-based self-help program targeting sexual problems and fertility-related distress in young adults diagnosed with cancer. The Fex-Can intervention has been developed in close cooperation with young patients with cancer and significant others [19], and has previously been evaluated as to its feasibility with results indicating it to be acceptable and safe to use [24]. The current study protocol describes the procedures for the clinical trial of the Fex-Can intervention.

The design of the trial has several significant strengths. First, a key strength is the RCT design which is the “gold standard” in clinical research and allows firm conclusions with regard to the efficacy of the intervention. Also, as the trial has a two parallel-arm design, both versions of the intervention, the Fex-Can Sex (targeting sexual problems) and the Fex-Can Fertility (targeting fertility-related distress) will be evaluated. Another significant strength of the design is the use of both short- and long-term follow-up assessments. In the previous literature, long-term evaluations (> 2 years) of web-based interventions have rarely been conducted [34]. The long follow-up period in this project, 20 and 44 months after the intervention, will thus enable conclusions with regard to a potential lasting effect of the intervention. Furthermore, the use of national quality registers allows identification of the total population of young adults diagnosed with the cancer diagnoses selected for the project, as well as provision of clinical data of high quality. The quality of the survey data is also high due to the use of validated instruments. Lastly, the use of secondary outcomes will allow investigation of potential additional effects, as well as of interactions with other important processes related to the outcomes targeted by the intervention, and will therefore increase the clinical relevance of conclusions.

Some potential weaknesses of the trial should also be mentioned. First, as prevalence rates for sexual problems and fertility-related distress following different cancer diagnoses have not yet been established, there are some uncertainties with regard to inclusion and retention rate. This may impact on the time needed to include a sufficient number of participants to reach adequate statistical power. Also, the design with no active treatment for the control-group aside from standard care should be mentioned as it may imply a risk of general, rather than specific, effects by the intervention. Future studies should compare the effects of different interventions on these issues. Lastly, the cut-off values used for inclusion have not been evaluated previously, and the relevance of sexual problems and fertility-related distress at levels above the cut-offs used in this trial are yet to be determined.

In conclusion, the current study protocol describes the clinical trial of the Fex-Can intervention which is the first web-based intervention targeting sexual problems and fertility-related distress in young adults diagnosed with cancer. Should the Fex-Can intervention prove to be efficacious, in the short- and/or in the long-term, we will take the necessary steps to implement it in routine care for young adults diagnosed with cancer. Healthcare could thereby be provided with an easily accessible, cost-effective intervention to offer to young adults suffering from fertility-related distress or sexual problems. This intervention thus has the potential to advance health care, and to improve the reproductive and sexual health in young people diagnosed with cancer in the future.

## Appendix

**Table 2:** Items from the World Health Organization Trial Registration Data Set

Data category	Information
Primary registry and trial identifying number	ISRCTN ISRCTN36621459
Date of registration in primary registry	25 January 2016
Secondary identifying numbers	10.1186/ISRCTN36621459
Source(s) of monetary or material support	Karolinska Institutet
Primary sponsor	Cancer Research Foundations of Radiumhemmet Swedish Cancer Society Swedish Childhood Cancer Foundation Vårdal Foundation Swedish Research Council for Health, Working Life and Welfare Swedish Research Council
Secondary sponsor(s)	Doctoral School in Health Care Sciences at Karolinska Institutet
Contact for public queries	*Lena Wettergren*, PhD [+46 (0)8524836 50] [lena.wettergren@ki.se] *Claudia Lampic*, PhD [+ 46 (0)8524823 70] [claudia.lampic@ki.se]
Contact for scientific queries	*Lena Wettergren*, PhD Karolinska Institutet, Sweden
Public title	Fex-Can – Fertility and sexuality following cancer
Scientific title	Fex-Can – interventions to alleviate impact of cancer on fertility and sexuality among adolescents and young adults
Countries of recruitment	Sweden
Health condition(s) or problem(s) studied	Brain tumor, breast cancer, cervical cancer, lymphoma, ovarian cancer, and testicular cancer
Intervention(s)	The Fex-Can intervention consists of two programs, The Fex-Can Sex and the Fex-Can Fertility, targeting sexual dysfunction and fertility-related distress respectively. They are web-based self-help programs delivered over a period of 12 weeks.
Key inclusion and exclusion criteria	Ages eligible for study: 18–39 years at diagnosis Sexes eligible for study: both Accepts healthy volunteers: no Inclusion criteria: adult patient (≥ 18 years) approximately 18 months after being diagnosed with cancer. Reporting sexual dysfunction or a high level of fertility-related distress. Exclusion criteria: non-ability to read and/or understand Swedish language. Reporting poor health and/or significant cognitive impairment that hinder participation in the intervention.
Study type	Interventional Allocation: randomized Intervention model: parallel assignment in blocks stratified by sex and diagnosis. The process will be performed separately for the arms of the RCT (Fex-Can Sex and Fex-Can Fertility) Masking: not possible due to the nature of the intervention Primary purpose: Efficacy trial
Date of first enrolment	September 2017
Target sample size	256
Recruitment status	Recruiting
Primary outcome(s)	The primary outcome for the Fex-Can Sex program is the score (continuous measure) of the domain ‘Satisfaction with sex life’, a subscale of the Patient-Reported Outcomes Measurement Information System® Sexual Function and Satisfaction measure. The primary outcome of the Fex-Can Fertility will be the summary score of the Reproductive Concerns After Cancer (RCAC).
Key secondary outcomes	Additional domains of sexual function (for participants in the Fex-Can Sex) and fertility-related knowledge (for participants in the Fex-Can Fertility). In addition, body image, health-related quality of life, anxiety and depression, self-efficacy (related to sexuality or fertility) measures will be used as secondary outcomes in both arms.

## Abbreviations

BIS: Body image scale
Fex-Can Cohort: Fex-Can population-based cohort study
Fex-Can Fertility: Fex-Can fertility intervention
Fex-Can Sex: Fex-Can sexuality intervention
Fex-Can: Fertility and sexuality following cancer
GDPR: General data protection regulation
HADS: Hospital anxiety and depression scale
PROMIS: Patient-Reported Outcomes Measurement Information System®
RCAC: Reproductive concerns after cancer scale
RCT: Randomized controlled trial
SexFS: Sexual function and satisfaction measure
